# Health-Related Quality of Life in Children and Adolescents with Overweight, Obesity, and Severe Obesity: A Cross-Sectional Study

**DOI:** 10.1159/000529560

**Published:** 2023-02-09

**Authors:** Kelly G.H. van de Pas, Maartje A.P. de Krom, Bjorn Winkens, François M.H. van Dielen, Anita C.E. Vreugdenhil

**Affiliations:** ^a^Centre for Overweight Adolescent and Children's Healthcare (COACH), Department of Pediatrics, Maastricht University Medical Centre+, Maastricht, The Netherlands; ^b^Department of Surgery, Máxima Medical Center, Veldhoven, The Netherlands; ^c^NUTRIM School of Nutrition and Translational Research in Metabolism, Maastricht University, Maastricht, The Netherlands; ^d^Department of Methodology and Statistics, Care and Public Health Research Institute (CAPHRI), Maastricht University, Maastricht, The Netherlands

**Keywords:** Quality of life, Children, Overweight, Obesity, Severe obesity

## Abstract

**Introduction:**

Children and adolescents with overweight and obesity have an impaired health-related quality of life (HRQoL). However, it is unclear which of these children are most affected in their physical, psychological, and social functioning. Therefore, this study aimed to evaluate HRQoL in treatment-seeking children and adolescents with overweight, obesity, and severe obesity.

**Methods:**

A cross-sectional study was performed at the Centre for Overweight Adolescent and Children's Healthcare (COACH). Children and adolescents (8–17 years) with overweight, obesity, and severe obesity were included. The primary outcome was the self-reported HRQoL measured with the KIDSCREEN-27.

**Results:**

A total of 419 participants with overweight (*N* = 121), obesity (*N* = 182), and severe obesity (*N* = 116) were included. One-way ANOVA analysis showed that children and adolescents with severe obesity reported significantly lower physical well-being (41.25 ± 13.14) compared to those with overweight (47.91 ± 12.53; *p* < 0.001) and obesity (46.74 ± 11.93; *p* < 0.001). Furthermore, impaired psychological well-being was found in the group with severe obesity (45.14 ± 13.27) in comparison to the group with overweight (50.90 ± 9.48; *p* < 0.001) and obesity (49.71 ± 10.95; *p* = 0.002). Multivariable linear regression analysis, while correcting for age, sex, cardio metabolic health risk, and ethnicity, revealed similar results. Additionally, children and adolescents with severe obesity scored lower regarding autonomy and parent relation than those with overweight (*B* = 3.95; *p* = 0.009). In almost all groups and dimensions of the KIDSCREEN-27, caregivers scored lower compared to the children and adolescents themselves. Furthermore, a low child-caregiver agreement seemed to exist, especially in the children and adolescents with overweight.

**Conclusion:**

The HRQoL of treatment-seeking children and adolescents with overweight and obesity was most affected in children and adolescents with the most severe grade of obesity. Following these findings, lifestyle intervention programs targeting childhood obesity should be aware of this even more vulnerable group so that treatments can be tailored according to their needs.

## Introduction

Childhood overweight is recognized as one of the most complex and pressing health challenges that the world is facing today [1]. In 2021, the prevalence of overweight in Dutch children aged between 4 and 17 years was 12.3%, whereas 3.5% of the children had obesity [2]. Although the prevalence of childhood overweight has stabilized over time, the recent COVID-19 pandemic contributed to an increase of more than 3% in the number of children with obesity compared to the pre-pandemic period [3, 4, 5]. This is troublesome, as childhood overweight and obesity are associated with several life-threatening conditions including type 2 diabetes mellitus and fatty liver disease [6, 7].

Besides the physical consequences, childhood overweight and obesity can result in substantial psychosocial problems [8, 9, 10, 11, 12]. Children and adolescents with overweight and obesity have to deal with weight-related social stigmatizing, discrimination and bullying, leading to a negative self-image, body dissatisfaction, depressive and anxiety symptoms, and social isolation [10, 11, 12]. These aspects contribute to an impaired health-related quality of life (HRQoL) in children with overweight and obesity compared to peers with normal weight [8, 10, 13, 14, 15, 16, 17, 18, 19]. Schwimmer et al. [8] even found a similar HRQoL in children with obesity and children diagnosed with cancer. Taken together, these results highlight that childhood overweight has a substantial negative impact on daily functioning and well-being.

Although there is mounting evidence of an impaired HRQoL in children and adolescents with overweight and obesity, it is unknown which of these children have the lowest HRQoL and are mostly affected in their physical, psychological, and social functioning [8, 10, 13, 14, 15, 16, 17, 18, 19]. A cross-sectional study of 45 treatment-seeking Dutch adolescents observed lower physical functioning and comfort in adolescents with severe obesity compared to adolescents with overweight [20]. In addition to this study, it is important to further identify the children and adolescents whose HRQoL is most affected in order to tailor treatments accordingly. Especially as a previous study showed that a higher HRQoL can improve therapy compliance [21]. Furthermore, it is essential to use a validated questionnaire that is equally understandable for children and adolescents from different cultures, such as the KIDSCREEN-27 [22, 23]. Therefore, the primary aim of this study was to evaluate HRQoL in treatment-seeking children and adolescents with overweight, obesity, and severe obesity. It was hypothesized that children and adolescents with the most severe grade of obesity had the worst HRQoL. Besides this, the self-reported and caregiver-reported HRQoL scores were compared, and the child-caregiver agreement was evaluated.

## Materials and Methods

### Setting and Study Design

This cross-sectional study was performed within the setting of the Centre for Overweight Adolescent and Children's Healthcare (COACH) at the Maastricht University Medical Centre + (MUMC+). Within this setting, all children and adolescents with overweight, obesity, and severe obesity underwent a comprehensive pre-intervention assessment. The assessment aimed to exclude syndromic or endocrine conditions of the weight status, to evaluate the presence of obesity related comorbidities, and to determine the psychological well-being of the children and adolescents before the start of the COACH lifestyle intervention [24]. Based on the outcomes, several healthcare professionals including pediatricians, psychologists, and dieticians set up a patient-tailored lifestyle intervention program for each child and adolescent focusing on durable lifestyle changes.

The present study was performed according to the guidelines of the Declaration of Helsinki and approval was given by the Medical Ethical Committee of the MUMC+ (METC-number 13-4-130). The study is registered at ClinicalTrial.gov as NCT02091544. Informed consent was obtained from all parents/legal guardians and children aged 12 years and older.

### Study Participants

All children and adolescents who received an assessment prior to the start of the COACH lifestyle intervention between May 2014 and December 2021 were considered for inclusion in this study. Children and adolescents aged 8–17 years old with overweight, obesity, and severe obesity were eligible to participate. Children below the age of 8 were not considered for inclusion since the questionnaire measuring our primary outcome was exclusively validated in children aged 8 years and older. Children and adolescents were excluded from the analyses when self-reported HRQoL data were missing.

### Participant Characteristics

Trained staff performed height and weight measurements during pre-intervention assessment. BMI was determined as the weight in kilograms divided by the height in meters squared. BMI z-scores were calculated using the Growth analyzer (GrowthAnalyser, VE). Participants were divided into the group with overweight, obesity, and severe obesity according to the International Obesity Task Force criteria, which are comparable to a BMI of ≥25 kg/m^2^, ≥30 kg/m^2^, and ≥35 kg/m^2^ in adults [25]. Children were defined as aged 8–11 years, while adolescents were classified as aged 12–17 years [26]. Ethnicity was classified as Dutch, Western, and Non-Western according to the Dutch Central Agency for Statistics [27]. Besides this, parent's educational level was divided into low, moderate, and high [28]. Low parental educational attainment was assigned when both parents had completed less than secondary vocational education. A high level of parental education was assigned when both parents had completed more than secondary vocational education. In other cases, the parents' educational level was considered moderate. At last, several medical conditions, including chronic/congenital somatic disease (e.g., diabetes mellitus type 1 and asthma), developmental disorders (e.g., Attention Deficit Hyperactivity Disorder and autism spectrum disorders), cancer, and mental health disorders (e.g., anxiety disorders and depression) were noted [29].

### Health-Related Quality of Life

The primary outcome was the HRQoL measured with the Dutch version of the validated KIDSCREEN-27 questionnaire. This instrument includes 27 items covering five different HRQoL dimensions. The physical well-being dimension (five questions) referred to the general health of a child or adolescent and their level of physical activity, fitness, and energy. The psychological well-being dimension included seven questions regarding positive emotions, satisfaction with life, level of self-esteem and the presence of loneliness and/or sadness. The third dimension, autonomy and parent relations (seven questions), explored the relationship between the child or adolescent and their parents or caregivers, level of autonomy, and financial sources. The relationship between the child or adolescent and their peers and the support they experienced from them is discovered in the social support and peers dimension (four questions). The last dimension referred to the school environment which includes four questions regarding the perception of the child or adolescent on their own cognitive capacity and skills, the relationship with teachers and their own feelings about school [26]. A Likert scale with five options, ranging from 1 = “not at all/never” to 5 = “all the way/always” was used [26]. Some items had to be reversed when scoring the questionnaire. Both the participant and caregiver separately completed the questionnaire at home or in the hospital. A scoring algorithm was used to convert the raw scores into T-scores [26]. If at least one item per dimension was left unanswered, the overall dimension score was identified as missing. Higher T-scores reflect better HRQoL.

### Cardio Metabolic Health Risk

During the clinical pre-intervention assessment, fasting levels of serum triglycerides, high-density lipoprotein (HDL), and glucose were measured. These levels combined with the average of at least three blood pressure measurements were used to determine the cardio metabolic health risk according to the Dutch Care Standard Obesity which was based on a consensus of the International Diabetes Federation [30, 31]. Triglycerides ≥1.7 mmol/L, HDL <1.03 mmol/L or HDL <1.29 mmol/L (females >16 years of age), and glucose ≥5.6 mmol/L were considered as abnormal values [30, 31]. According to a recent guideline, blood pressure was elevated when systolic and/or diastolic blood pressure ≥90th percentile for sex, age, and height [32]. All these values were gathered, and when participants (≥10 years of age) had at least one abnormal value, they were identified as having an increased cardio metabolic health risk. In line with the Dutch Care Standard Obesity, children <10 years of age were not considered as having an increased cardio metabolic health risk. When two or more values were missing, the cardio metabolic health risk was identified as missing.

### Statistical Analysis

Statistical analyses were performed with IBM SPSS Statistics, version 26.0, Armonk, NY. Two-sided *p* values ≤0.05 were considered statistically significant. First of all, the numerical baseline characteristics were checked for homoscedasticity with the homogeneity of variances test, and normality using P-P plots and histograms. If the assumptions were not violated, the numerical baseline characteristics were analyzed using one-way ANOVA tests. Categorical baseline characteristics were analyzed using χ^2^ or Fisher-Freeman-Halton Exact tests. Both self-reported and caregiver-reported T-scores for the HRQoL data were presented. The self-reported and caregiver-reported T-scores were checked for normality using P-P plots and histograms; homoscedasticity was controlled by the homogeneity of variances test and linearity using scatterplots. Outliers were checked using Cook's distance, a distance >1 indicated an influential outlier. Multicollinearity was assessed by the variance inflation factor, and a variance inflation factor >10 indicated a multicollinearity problem. If the assumptions were not violated, one-way ANOVA and multivariable linear regression analyses were performed to compare the self-reported HRQoL dimensions between the group with overweight, obesity, and severe obesity. In case of a *p* ≤ 0.05 in the one-way ANOVA analyses, post hoc tests were conducted using Tukey's method to correct for multiple testing. Results were checked for important patient perceived differences in HRQoL (clinical relevance). Important patient perceived differences in HRQoL between groups were earlier defined as half a standard deviation (SD) of the total group score [33]. In the multivariable linear regression analyses, a top-down procedure was used to obtain the final model. First, interactions between age*weight category and sex*weight category were assessed as the effect of weight category on HRQoL might depend on age and sex. In case of a significant interaction, the effect of weight category was presented for different values of that effect modifier. If the interactions were not significant, the interactions were removed from the model and the main effect of weight category was given. In all models, corrections were made for participants' age, sex, the cardio metabolic health risk, and ethnicity according to literature [8]. The group with severe obesity was considered as the reference category. As a sensitivity analysis, the significance level was set at 0.01 to account for multiple testing in the multivariable linear regression analyses. The difference between self-reported and caregiver-reported mean T-scores was evaluated using paired samples *t* tests and the correlation between these scores was investigated using Pearson correlations. Furthermore, child-caregiver agreement was evaluated using Bland-Altman plots. In the Bland-Altman plots, the Y-axis represents the difference in self-reported and caregiver-reported T-score, while the X-axis represents the mean of the participants' and caregiver T-score [34].

## Results

**Fig. 1:**
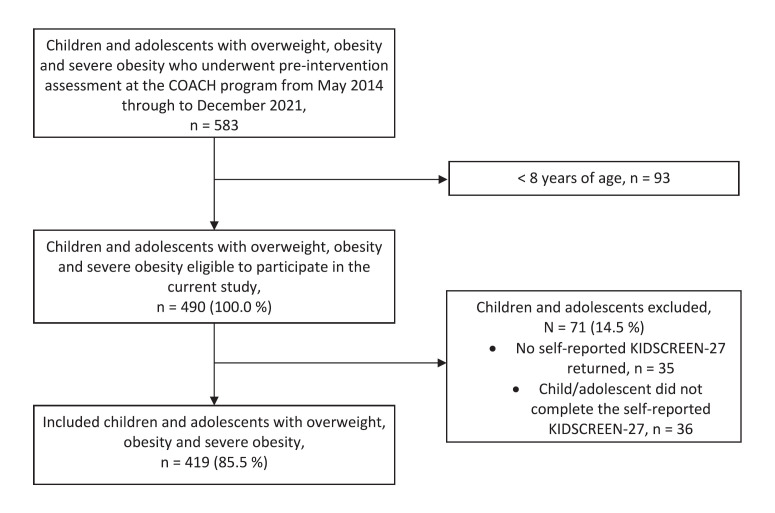
Flow diagram of the inclusion of children and adolescents with overweight, obesity, and severe obesity.

A total of 583 children and adolescents with overweight, obesity, and severe obesity underwent pre-intervention assessment before the start of the COACH lifestyle intervention. From these, 490 children and adolescents were eligible to participate since they were aged 8–17 years old (Fig. 1). After excluding 71 children and adolescents (14.5%) who did not return or complete the self-reported KIDSCREEN-27 questionnaires, 419 children and adolescents (85.5%) were included. The most frequently mentioned reasons for not completing or returning the KIDSCREEN-27 were misunderstanding of the questionnaire or a language barrier. The response rates between the group with overweight, obesity, and severe obesity were comparable; the response rates were 88.9%, 84.7%, and 83.5%, respectively. The only differences between the 419 included participants and the 71 excluded participants were found in the higher prevalence of non-Western ethnicity and mental health disorders among the excluded children and adolescents (data not shown).

### Participant Characteristics

The characteristics of the included children and adolescents with overweight, obesity, and severe obesity are presented in Table 1. The children and adolescents with severe obesity were significantly older compared to those with overweight and obesity (*p* = 0.001 and *p* = 0.015). Furthermore, there were significant differences among the three groups regarding parent's education.

### Self-Reported HRQoL

**Fig. 2:**
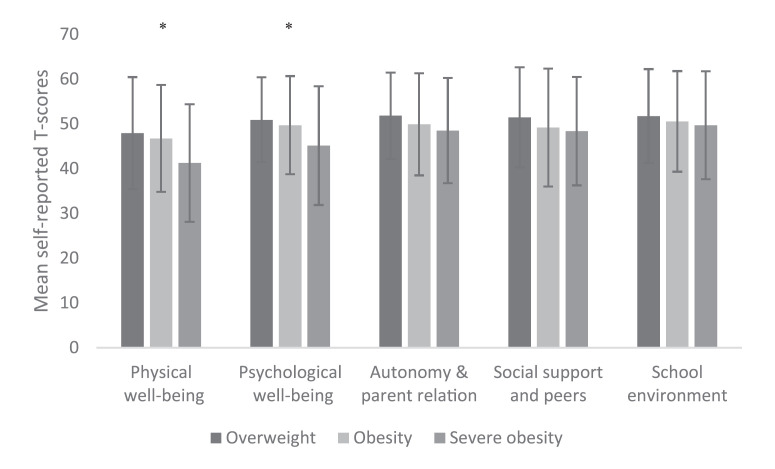
Mean self-reported T-scores (±SD) on KIDSCREEN-27 in the three different weight categories. SD, standard deviation. Physical wellbeing: overweight *n*= 120; obesity *n* = 178; severe obesity *n*= 116. Psychological wellbeing: overweight *n*= 121; obesity n=181; severe obesity *n*= 115. Autonomy & parent relation: overweight *n*= 120; obesity *n* = 180; severe obesity *n* = 114. Social support and peers: overweight *n* = 120; obesity *n*= 180; severe obesity *n*= 114. School environment: overweight *n* = 119; obesity *n* = 180; severe obesity *n*= 114. * Statistically significant difference between participants with severe obesity and those with overweight and obesity (*p* ≤ 0.05).

Children and adolescents with severe obesity scored on average lower in all dimensions of the self-reported KIDSSCREEN-27 compared to children and adolescents with overweight or obesity (Fig. 2). One-way ANOVA revealed significant differences between groups in the physical and psychological well-being dimensions (both *p* < 0.001). Children and adolescents with severe obesity reported significantly lower physical well-being (41.25 ± 13.14) compared to those with overweight (47.91 ± 12.53; *p* < 0.001) and obesity (46.74 ± 11.93; *p* < 0.001). Furthermore, impaired psychological well-being was found in the group with severe obesity (45.14 ± 13.27) in comparison to the group with overweight (50.90 ± 9.48; *p* < 0.001) and obesity (49.71 ± 10.95; *p* = 0.002). The differences in physical and psychological well-being between the children and adolescents with overweight and severe obesity were also clinically relevant (>0.5 SD).

Multivariable linear regression analysis of self-reported T-scores on the KIDSCREEN-27 dimensions between children and adolescents with overweight, obesity, and severe obesity, while adjusting for age, sex, cardio metabolic health risk, and ethnicity, revealed similar results (Table 2). The children and adolescents with severe obesity reported significantly lower physical and psychological well-being compared to the group with overweight and obesity. Additionally, participants with severe obesity scored significantly lower regarding autonomy and parent relation than those with overweight (*B* = 3.95; *p* = 0.009). In the sensitivity analysis (using a significance level of 0.01 instead of 0.05 to adjust for multiple testing), the same conclusions were drawn as all significant *p* values were below 0.01.

### Self-Reported versus Caregiver-Reported HRQoL

**Fig. 3:**
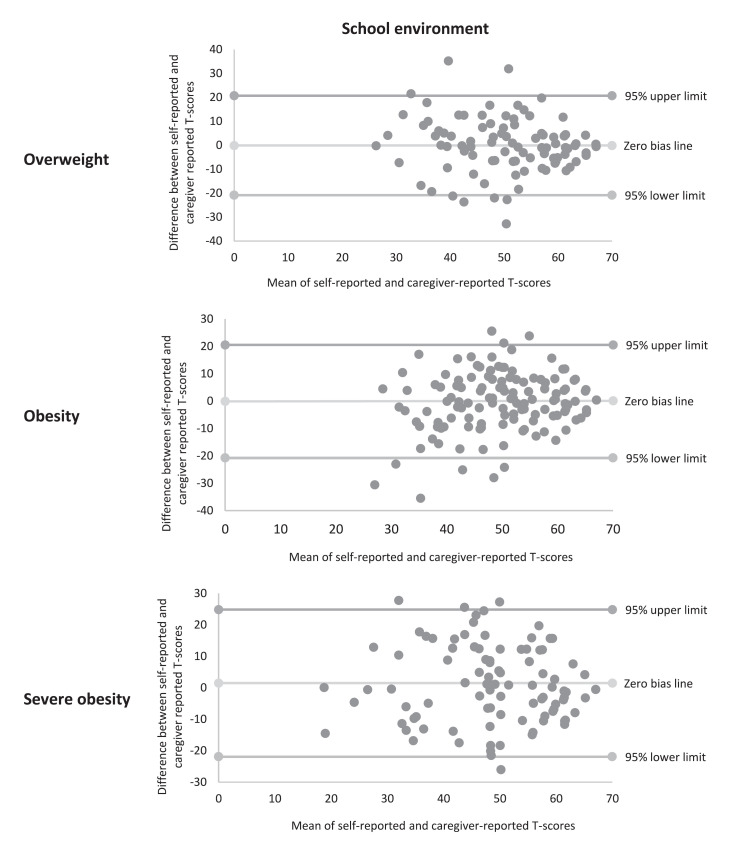
Bland-Altman plots of school environment dimension stratified by weight category. Difference between self-reported (ST) and caregiver reported (CT) T-scores; ST-CT. Mean of self-reported and caregiver-reported T-scores (ST + CT)/2.

The self-reported compared to the caregiver-reported mean T-scores on the KIDSCREEN-27 are presented in Table 3. The mean caregiver scores were lower on almost all dimensions of the KIDSCREEN-27 compared to the mean scores of children and adolescents with overweight, obesity, and severe obesity themselves. In the physical well-being dimension, the mean caregiver-reported T-scores were significantly lower compared to the self-reported T-scores in all three weight categories. Furthermore, the mean caregiver-reported T-scores were significantly lower in four out of five KIDSCREEN-27 dimensions in the group with overweight. In the group with obesity, significant differences between the caregiver and self-reported mean T-scores were found in the physical and psychological well-being dimensions, whereas in the group with severe obesity a significant difference was only found in the physical well-being dimension. Pearson correlation coefficients between self-reported and caregiver-reported T-scores in the KIDSCREEN-27 dimensions ranged from 0.22 to 0.71. Individual child-caregiver agreement was evaluated using Bland-Altman plots (Fig. 3; Supporting Information online suppl. Fig. S1; see www.karger.com/doi/10.1159/000529560 for all online suppl. material). The upper and lower limits of the Bland-Altman plots were relatively broad in all dimensions and weight categories, indicating a low agreement on individual level. Visual interpretation suggests that there is more agreement when the T-scores were higher, as the dots are situated closer to the zero-bias line.

## Discussion

The psychosocial consequences of childhood overweight and obesity are severe, resulting in a negative self-image, body dissatisfaction, and social isolation which leads to an impaired HRQoL [10, 11, 12]. This study identified that treatment-seeking children and adolescents with the most severe grade of obesity were the most affected in their HRQoL, mainly in the physical, psychological, and autonomy and parent-related dimensions. These findings were also clinically relevant as the children and adolescents with severe obesity had >0.5 SD lower HRQoL scores compared to children and adolescents with overweight and to a Dutch reference population [35]. Additionally, caregivers felt that their children's HRQoL was worse than the children and adolescents experienced themselves. Besides this, there were large individual discrepancies between caregivers and children, assuming low agreement.

The physical well-being dimension is one of the HRQoL dimensions that is most affected by the degree of overweight and obesity in children and adolescents. The pooled analyses of a systematic review revealed a strong inverse relation between physical well-being and BMI [36]. Moreover, a recently conducted study among treatment-seeking Dutch adolescents with overweight and obesity observed a lower physical functioning and comfort on the CHQ Child Form 87 and Impact of Weight on Quality of Life-Kids questionnaires in adolescents with obesity grade 3 compared to those with overweight [20]. In line with these studies, the present study found a clinically relevant lower physical well-being in children and adolescents with severe obesity compared to those with overweight, and even in comparison to those with obesity. In clinical practice, a lower physical well-being resembles less energy, physical activity, and health in general. According to these findings, lifestyle interventions targeting childhood obesity should take into account that children and adolescents with the most severe grade of obesity are less physically active and need extra attention to improve their physical activity. Particularly as a previous study found that long-term changes in physical activity, and not in BMI, explained 30% of the variation in HRQoL [37].

In contrast to physical well-being, there is less consensus in literature regarding weight-related psychological well-being [10, 36]. The present study found a clinically relevant lower psychological well-being in the children and adolescents with severe obesity, indicating that these children are less satisfied with themselves and are more often lonely and sad. Remarkably, sex as well as age were not significantly related to psychological well-being in our study. This is in contrast with some other reports, which revealed that girls might be more vulnerable to psychological complaints related to their weight status because of higher levels of body dissatisfaction and lower levels of body esteem [38, 39]. Besides this, older children might be more aware of their appearance and social limitations due to their weight and therefore have a lower psychological well-being [20]. These conflicting results regarding weight, sex- and age-related psychological well-being might be due to different study populations (treatment-seeking or population based), various degrees of overweight and different questionnaires used to evaluate psychological well-being. Continued efforts are needed to identify determinants that influence psychological well-being, as well as the other quality of life dimensions in children and adolescents with overweight and obesity.

Due to the increased prevalence of chronic somatic and mental disorders among children and adolescents, monitoring of HRQoL has become an important topic in health care [40]. A large population-based study performed in the Netherlands reported a lower HRQoL among children and adolescents with chronic health problems (e.g., asthma, eczema, ADHD, dyslexia, and migraine) compared to those without a chronic condition [41]. Previous research also found an impaired HRQoL in youth with overweight and obesity compared to youth with a normal weight [8, 10, 13, 14, 15, 16, 17, 18, 19]. Our results revealed an even worse HRQoL in children and adolescents with the most severe grade of obesity. Furthermore, a recent meta-analysis estimated the magnitude of the HRQoL impairments measured with the KIDSCREEN questionnaire in children and adolescents with chronic health problems. The largest impairments in HRQoL were found in children with malignant neoplasms, endocrine, and metabolic diseases (e.g., obesity), diseases of the nervous system, congenital malformations, and chronic pain [42]. This indicates that childhood obesity is a severe chronic disease with a huge impact on HRQoL and that its psychosocial consequences should be given equal attention and support as other chronic diseases.

When assessing the HRQoL of children and adolescents with overweight and obesity, it is recommended to use both self-reported and caregiver-reported scores to evaluate HRQoL comprehensively [20, 43]. Most previous studies focusing on the HRQoL of children and adolescents with overweight and obesity found lower caregiver-reported HRQoL scores compared to self-reported HRQoL scores, which is in line with our results [10, 36]. One interesting finding of the current study was that low child-caregiver agreement seemed to exist in the children and adolescents, especially in the ones with overweight. Children and adolescents with overweight might experience fewer obesity related complaints or do not relate their complaints or well-being to their weight status, while caregivers are more aware of the consequences of the weight status of their child [44]. In children and adolescents with severe obesity, the link between symptoms and weight status might be more apparent for both children and caregivers. Following these findings, one should be aware of the discrepancies between how parents and children with overweight and obesity experience quality of life, especially in children and adolescents with overweight.

The present study has some limitations that should be acknowledged. First, the use of a questionnaire in this study may have led to recall bias. Second, it is questionable whether our results can be generalized to all children and adolescents with overweight and obesity since our study population consisted of treatment-seeking children and adolescents with overweight, obesity, and severe obesity. The HRQoL in our study population might therefore be lower compared to non-treatment-seeking children and adolescents [45]. Finally, due to the cross-sectional design of this study, no conclusions can be drawn regarding the causal relationship between the degree of overweight and obesity and an impaired HRQoL.

## Conclusion

The HRQoL of treatment-seeking children and adolescents with overweight and obesity was most affected in the children and adolescents with the most severe grade of obesity, especially in the physical and psychological dimensions. Following these findings, lifestyle intervention programs targeting childhood obesity should be aware of this even more vulnerable group so that treatments can be tailored according to their needs. Subsequently, future longitudinal studies should focus on the effect of these programs on the HRQoL of children and adolescents with various degrees of overweight and obesity. Additionally, attention must be caught to discrepancies in HRQoL between children, adolescents, and their caregivers, especially in the group with overweight.

## Statement of Ethics

This study protocol was reviewed and approved by the Medical Ethical Committee of the MUMC+, METC-number 13-4-130. Written informed consent was obtained from all the participants and their parents or legal guardians where appropriate.

## Conflict of Interest Statement

The authors have no conflicts of interest to declare.

## Funding Sources

No funding was received for this study.

## Author Contributions

K.G.H.P., M.A.P.K., and A.C.E.V. designed the study; K.G.H.P. and M.A.P.K. collected and analyzed the data; B.W. helped with the statistical analyses; K.G.H.P., M.A.P.K., B.W., F.M.H.D. and A.C.E.V. were involved in writing the paper and had final approval of the submitted and published versions.

## Data Availability Statement

All data generated or analyzed during this study are included in this article and its online supplementary material files. Further inquiries can be directed to the corresponding author.

## Supplementary Material

Supplementary data

## Figures and Tables

**Table 1:** Participant characteristics of the included children and adolescents

	Total *n* = 419	Overweight *n* = 121 (28.9%)	Obesity *n* = 182 (43.4%)	Severe obesity *n* = 116 (27.7%)	*p* value
Age, years (mean ± SD)	12.93±2.55	12.47±2.43	12.79±2.54	13.62±2.55	**0.001** *

Age category, *n* (%)	419	121	182	116	**0.002**
Children	174 (41.5)	61 (50.4)	80 (44.0)	33 (28.4)	
Adolescents	245 (58.5)	60 (49.6)	102 (56.0)	83 (71.6)	

Sex, *n* (%)	419	121	182	116	0.707
Female	210 (50.1)	63 (52.1)	87 (47.8)	60 (51.7)	
Male	209 (49.9)	58 (47.9)	95 (52.2)	56 (48.3)	

BMI, kg/m^2^ (mean ± SD)	29.81±6.21	24.52±2.17	28.79±2.99	36.91±6.26	**<0.001** **

BMI z-score (mean ± SD)	3.16±0.69	2.38±0.34	3.17±0.29	3.98±0.41	**<0.001** **

Cardiometabolic health risk^a^, *n* (%)	412	121	180	111	0.266
Yes	174 (42.2)	45 (37.2)	76 (42.2)	53 (47.7)	
No	238 (57.8)	76 (62.8)	104 (57.8)	58 (52.3)	

Ethnicity^b^, *n* (%)	417	121	180	116	0.693
Dutch	289 (69.3)	90 (74.4)	121 (67.2)	78 (67.2)	
Western	34 (8.2)	8 (6.6)	15 (8.3)	11 (9.5)	
Non-western	94 (22.5)	23 (19.0)	44 (24.4)	27 (23.3)	

Parent's education^b^, *n* (%)	370	106	157	107	**<0.001**
Low	166 (44.9)	37 (34.9)	67 (42.7)	62 (57.9)	
Moderate	160 (43.2)	47 (44.3)	75 (47.8)	38 (35.5)	
High	44 (11.9)	22 (20.8)	15 (9.6)	7 (6.5)	

Family composition^ *n* (%)	418	121	181	116	0.424
Married	225 (53.8)	73 (60.3)	93 (51.4)	59 (50.9)	
Divorced	176 (42.1)	43 (35.5)	82 (45.3)	51 (44.0)	
Other	17 (4.1)	5 (4.1)	6 (3.3)	6 (5.2)	

Medical history, *n* (%)	419	121	182	116	
Chronic/congenital somatic disease 66 (15.8)	17 (14.0)	26 (14.3)	23 (19.8)	0.366
Developmental disorder	78 (18.6)	24 (19.8)	28 (15.4)	26 (22.4)	0.290
Cancer^c^	3 (0.7)	0 (0)	1 (0.5)	2 (1.7)	0.361
Mental health disorder^c^	10 (2.4)	1 (0.8)	5 (2.7)	4 (3.4)	0.396

BMI, body mass index; SD, standard deviation; N, number. Statistically significant differences (p < 0.05) are highlighted in boldface.

a Triglycerides >1.7 mmol/L, HDL-cholesterol <1.03 mmol/L or HDL <1.29 (for females >16 years of age), fasting glucose >5.6 mmol/L [30, 31], or systolic and/or diastolic blood pressure >90th percentile for sex, age, and height [32].

b According to the Dutch Central Agency for Statistics [27, 28].

c Fisher-Freeman-Halton Exact test.

* Statistically significant difference between participants with severe obesity and those with overweight and obesity.

** Statistically significant difference between all three weight status categories.

**Table 2:** Multivariable linear regression analysis of self-reported T-scores on the KIDSCREEN-27 dimensions between children and adolescents with overweight, obesity and severe obesity, while correcting for age, sex, cardio metabolic health risk, and ethnicity

	Physical well-being			Psychological well-being			Autonomy and parent relation			Social support and peers			School environment		
	*B(SE)*	β	p value	*B(SE)*	β	p value	*B(SE)*	β	p value	*B(SE)*	β	p value	*B(SE)*	β	p value
Overweight	6.46(1.67)	0.23	<0.001	5.70	0.23	<0.001	3.95	0.16	0.009	3.22	0.12	0.055	2.61	0.11	0.089
Obesity	5.13(1.52)	0.20	<0.001	4.28(1.39)	0.19	0.002	1.62(1.37)	0.07	0.237	0.73(1.53)	0.03	0.632	1.08(1.39)	0.05	0.440
Severe obesity	Ref			Ref			Ref			Ref			Ref		
Age	−0.39 (0.26)	−0.08	0.130	−0.34 (0.23)	−0.08	0.150	0.16(0.23)	0.04	0.500	−0.22 (0.26)	−0.05	0.389	0.29 (0.23)	0.07	0.219
Boys	−2.13(1.25)	−0.08		−0.01 (1.14)	0.00	0.993	−0.51 (1.12)	−0.02	0.654	−0.59(1.25)	−0.02	0.636	−1.13(1.14)	−0.05	0.322
Girls	Ref			Ref			Ref			Ref			Ref		
Cardiometabolic health risk	−0.68	−0.03	0.601	0.75	0.03	0.529	1.50(1.17)	0.07	0.199	1.03(1.30)	0.04	0.430	0.30(1.19)	0.01	0.799
No cardiometabolic health risk Ref			Ref			Ref			Ref			Ref		
Western ethnicity	2.20 (2.26)	0.05	0.332	−1.44(2.06)	−0.04	0.485	0.67 (2.08)	0.02	0.747	1.13(2.27)	0.03	0.617	−0.89 (2.06)	−0.02	0.668
Non-western ethnicity	2.20(1.51)	0.07	0.146	1.22(1.38)	0.04	0.378	0.81 (1.35)	0.03	0.548	1.27(1.52)	0.04	0.403	1.95(1.38)	0.07	0.159
Dutch ethnicity	Ref			Ref			Ref			Ref			Ref		

B, unstandardized regression coefficient indicating the association between this variable and the outcome; SE, standard error; p, standardized regression coefficient; ref, reference category. Statistically significant differences (p < 0.05) are highlighted in boldface.

**Table 3:** Difference and correlation between self-reported and caregiver-reported T-scores on the KIDSCREEN-27 stratified by weight categories

KIDSCREEN-27 T-score	Overweight					Obesity					Severe obesity				
	*N^a^*	mean self-reported (±SD)	mean caregiver-reported (±SD)	p value	*r*	*N^a^*	mean self-reported (±SD)	mean caregiver-reported (±SD)	p value	*r*	*N^a^*	mean self-reported (±SD)	mean caregiver-reported (±SD)	p value	*r*
Physical well-being	112	47.68±12.77	43.36±15.05	**<0.001**	**0.71**	165	46.49±11.94	41.12±12.54	**<0.001**	**0.51**	107	41.12±13.35	37.06±13.28	**0.001**	**0.61**
Psychological well-being	111	51.25±9.40	48.45±11.41	**0.005**	**0.52**	169	49.61 ±10.93	46.65±11.12	**0.002**	**0.37**	105	45.80±13.09	44.07±13.81	0.140	**0.61**
Autonomy and parent relation	110	51.76±9.66	48.68±9.73	**0.004**	**0.37**	166	49.88±11.57	49.81 ±9.95	0.944	**0.22**	103	48.86±12.08	48.47±12.64	0.773	**0.38**
Social support and peers	111	52.32±10.46	49.23±11.95	**0.005**	**0.49**	164	49.01 ±13.45	47.02±13.63	0.088	**0.40**	103	48.10±12.30	46.39±12.56	0.211	**0.39**
School environment	108	51.57±10.61	51.66±11.69	0.924	**0.55**	168	50.58±11.26	50.68±9.67	0.900	**0.50**	104	49.62±11.96	48.15±12.35	0.212	**0.52**

SD, standard deviation; N, number; r, Pearson correlation between self-reported and caregiver-reported T-scores. Statistical significance is highlighted in boldface (p< 0.05).

a Number of participants that both had self- reported and caregiver-reported T-scores on that dimension.
