# Supplementary material for: Health-Related Quality of Life in Children and Adolescents with Overweight, Obesity, and Severe Obesity: A Cross-Sectional Study
Source: Obes Facts. 2023 Feb 9;16(3):282–92. doi: 10.1159/000529560 (PMC10331158; doi:10.1159/000529560)
Supplement: Supplementary file 1 — Supplementary data [file ofa-0016-0282-s01.pdf]

Supporting information

Health-related quality of life in children and adolescents with overweight, obesity and severe obesity: a cross-sectional study

Physical wellbeing

Overweight

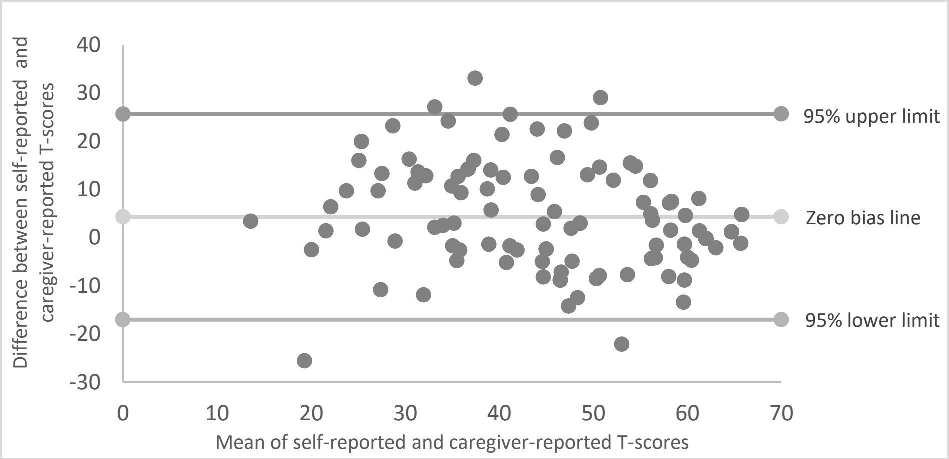

Obesity

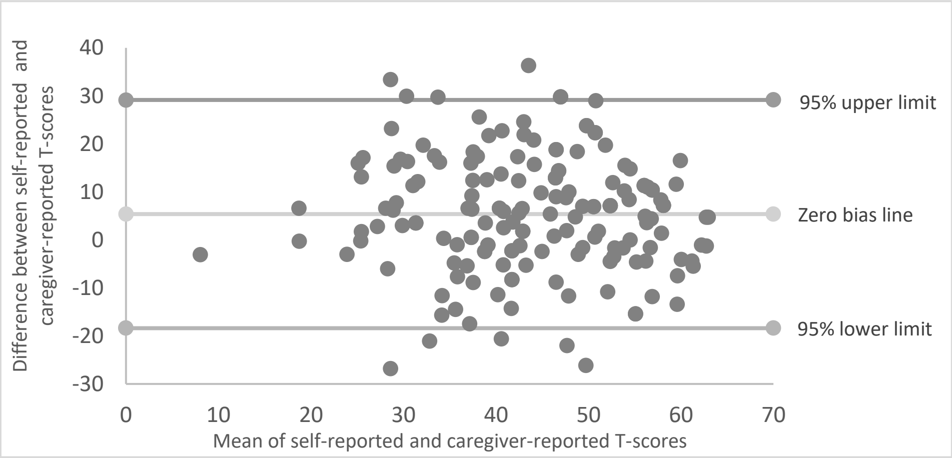

Severe obesity

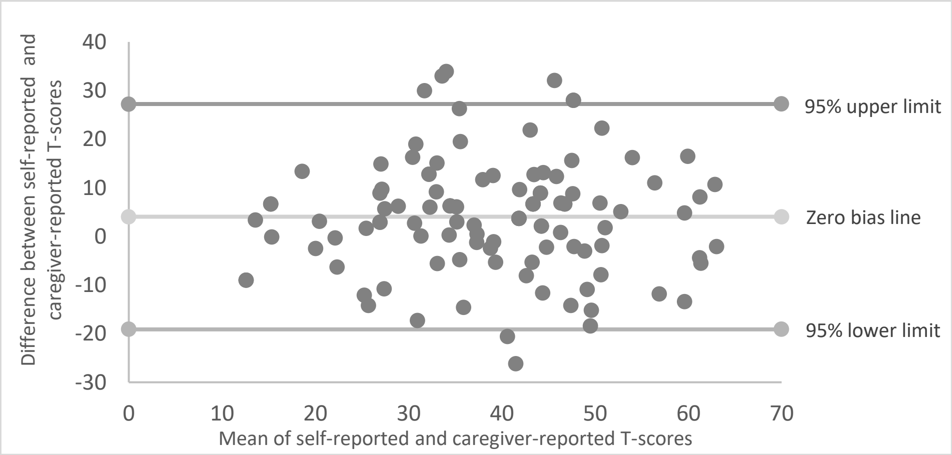

Psychological wellbeing

Overweight

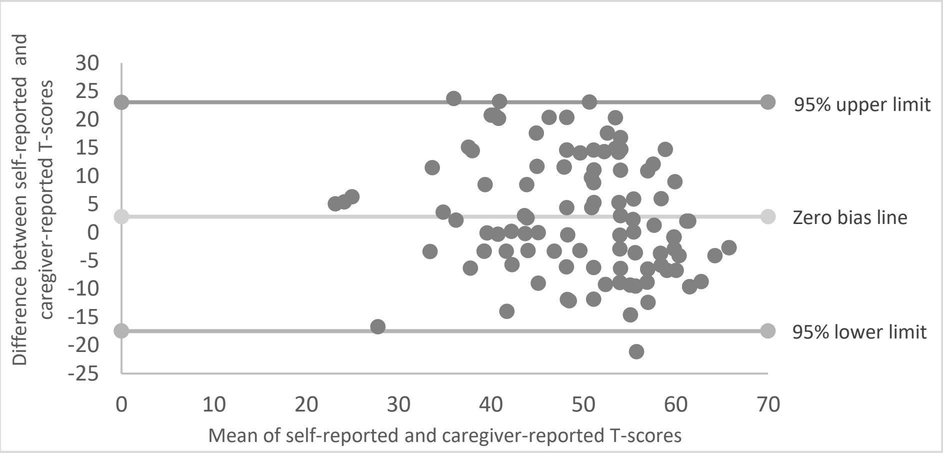

Obesity

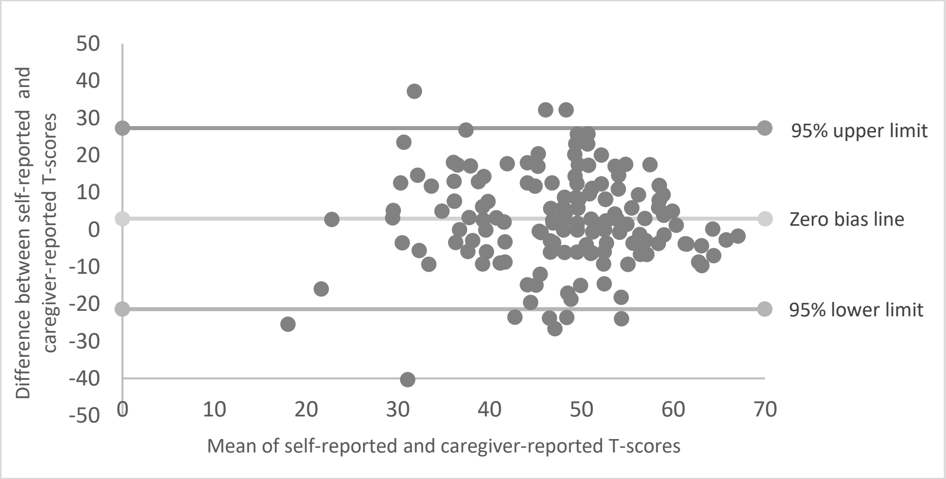

Severe obesity

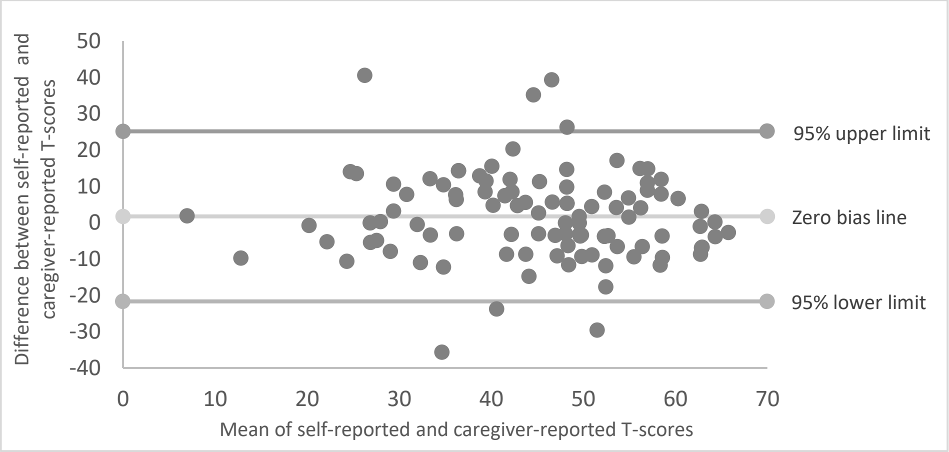

Autonomy & parent relation

Overweight

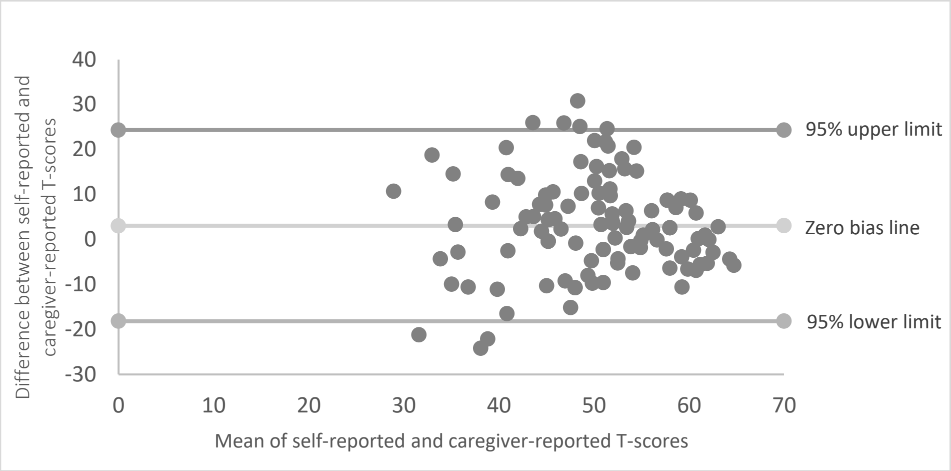

Obesity

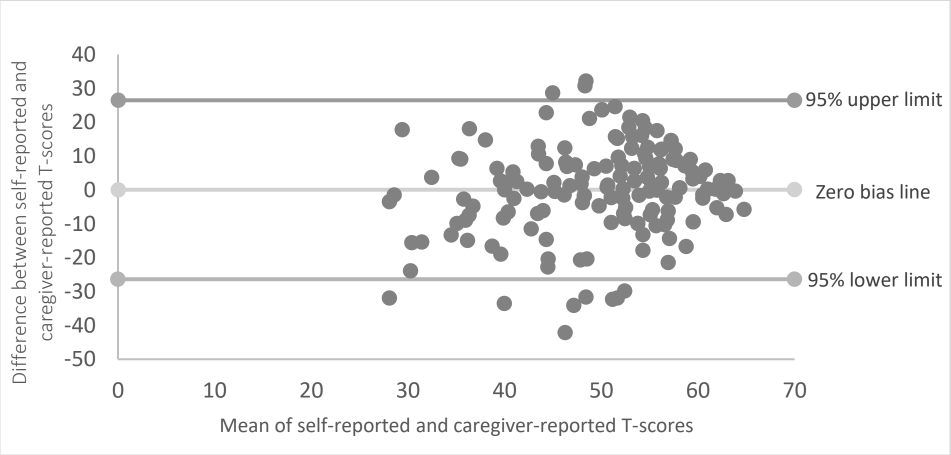

Severe obesity

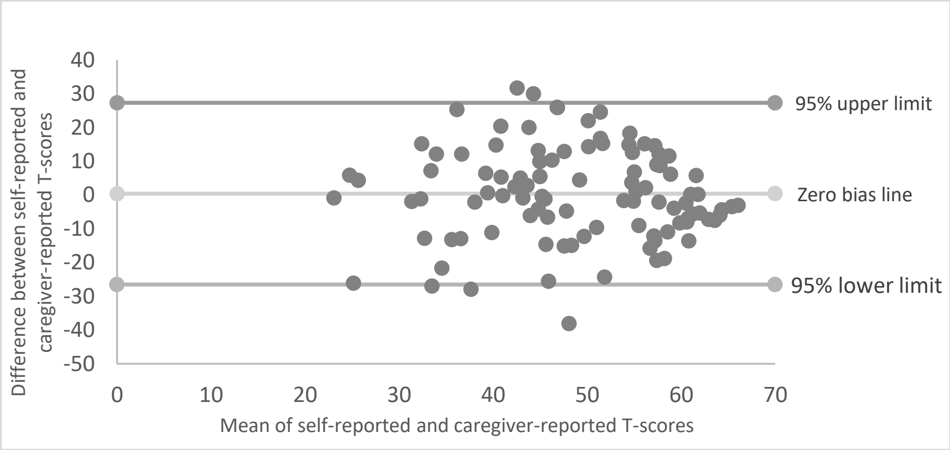

Social support and peers

Overweight

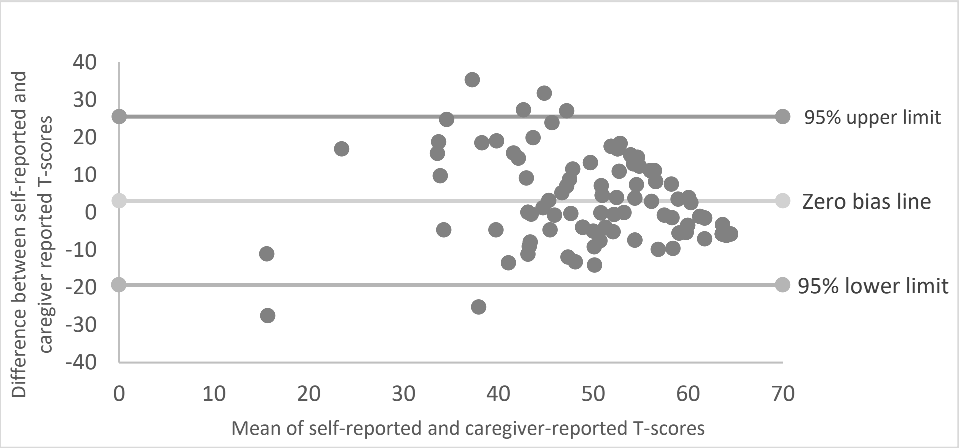

Obesity

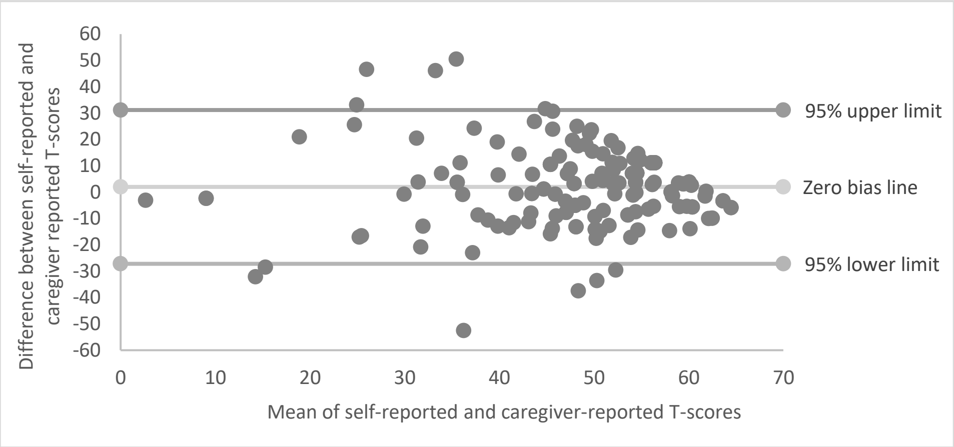

Severe obesity

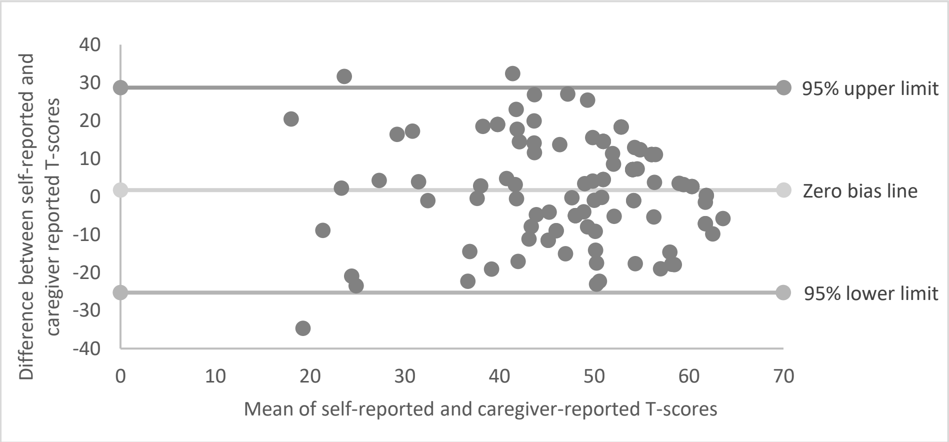

Figure S1. Bland-Altman plots of KIDSCREEN-27 dimensions (physical wellbeing, psychological wellbeing, autonomy & parent relation, social support and peers) stratified by weight category.
Difference between self-reported (ST) and caregiver reported (CT) T-scores; ST-CT
Mean of self-reported and caregiver reported T-scores; (ST+CT)/2
